# Ethnopharmacological Effects of *Urtica dioica*, *Matricaria chamomilla*, and *Murraya koenigii* on Rotenone-Exposed *D. melanogaster*: An Attenuation of Cellular, Biochemical, and Organismal Markers

**DOI:** 10.3390/antiox11081623

**Published:** 2022-08-21

**Authors:** Shabnam Shabir, Sumaira Yousuf, Sandeep Kumar Singh, Emanuel Vamanu, Mahendra P. Singh

**Affiliations:** 1 School of Bioengineering and Biosciences, Lovely Professional University, Phagwara 144411, India; 2 Indian Scientific Education and Technology Foundation, Lucknow 226002, India; 3 Faculty of Biotechnology, University of Agricultural Sciences and Veterinary Medicine, 011464 Bucharest, Romania

**Keywords:** antioxidants, acetylcholinesterase, 1,1-diphenyl-2-picrylhydrazyl, HPLC, medicinal plants, oxidative stress

## Abstract

Natural antioxidants derived from plants have been proven to have significant inhibitory effects on the free radicals of living organisms during actively metabolization. Excessive production of free radicals increases the risk of neurodegenerative diseases, such as Alzheimer’s disease, Parkinson’s disease, and motor sclerosis. This study aimed to compare the ethnopharmacological effects of *Urtica dioica* (UD), *Matricaria chamomilla* (MC), and *Murraya koenigii* (MK) on the amelioration of rotenone-induced toxicity in wild-type *Drosophila melanogaster* (Oregon R^+^) at biochemical, cellular, and behavioral levels. Phytoextracts were prepared from all three plants, i.e., UD, MC, and MK (aqueous and ethanolic fractions), and their bioactive compounds were evaluated using in vitro biochemical parameters (DPPH, ABTS, TPC, and TFC), UV-Vis, followed by FT-IR and HPLC. Third instar larvae and freshly eclosed flies were treated with 500 µM rotenone alone or in combination with UD, MC, and MK for 24 to 120 h. Following exposure, cytotoxicity (dye exclusion test), biochemical (protein estimation and acetylcholinesterase inhibition assays), and behavioral assays (climbing and jumping assays) were performed. Among all three plant extracts, MK exhibited the highest antioxidant properties due to the highest TPC, TFC, DPPH, and ABTS, followed by UD, then MC. The overall trend was MK > UD > MC. In this context, ethnopharmacological properties mimic the same effect in *Drosophila*, exhibiting significantly (*p* < 0.05) reduced cytotoxicity (trypan blue), improved biochemical parameters (proteotoxicity and AChE activity), and better behavioral parameters in the organisms cotreated with phyto extracts compared with rotenone. Conclusively, UV-Vis, FTIR, and HPLC analyses differentiated the plant extracts. The findings of this research may be beneficial in the use of select herbs as viable sources of phyto-ingredients that could be of interest in nutraceutical development and various clinical applications.

## 1. Introduction

The cellular redox status is determined by the balance between antioxidants and oxidants. The oxidative state of the cell is defined by an imbalance between these two, which can result in apoptosis or necrosis [1]. Reactive oxygen species (ROS) are primarily responsible for the high susceptibility of brain cells to oxidative stress [2]. Although oxygen is a relatively nonreactive substance, it can be metabolized in the body to create highly reactive free radicals, such as hydroxyl radicals (OH^−^), superoxide anions (O_2_^−^), and many other reactive species [3]. These free radical species contribute to the pathophysiology of many neurodegenerative diseases, including amyloidosis, α-synucleinopathies, aging, and TDP-43 proteinopathies, which are caused by the inhibition of acetylcholinesterase [4]. Detoxification is aided by cellular defense mechanisms that involve endogenous antioxidants and antioxidant enzymes such as superoxide dismutase, glutathione reductase, lipid peroxidase, glutathione, and catalase [5]. The deterioration of these defense mechanisms damages significant cell biomolecules (lipids, DNA, and proteins) and ultimately leads to cell death [6].

Currently, conventional medicines or therapeutic drugs (levodopa or dopaminergic agonists) have only been used to treat motor symptoms by restoring neurotransmitters [7]. The extended use of these drugs, however, can have negative effects, such as fatigue and other motor difficulties [8]. Considering these limitations, there is a need for novel natural neuroprotective agents to halt or slow the progression of various neurodegenerative disorders [9]. According to cumulative evidence, nutraceuticals and other phytochemicals have been shown to have neuroprotective effects and alleviate the symptoms of neurodegenerative diseases by activating the PI3K/Akt/Nrf2 pathway by scavenging free radicals [10]. Herbal biomolecules have been used as phytomedicines in healthcare industries since the emergence of civilization; according to the World Health Organization (WHO), phytomedicine is used as a primary source of therapy by ~79% of the world’s population [11]. *Urtica dioica* (UD), *Matricaria chamomilla* (MC), and *Murraya koenigii* (MK) have all been used as well-known cognition enhancers and nerve relaxants in the Ayurvedic medical system [12]. The phytochemicals of these three herbs, such as quercetin, polyphenols, alkaloids, reducing sugars, and vitamins, are known for their protective effects. UD has been extensively studied and has shown promising results in the treatment of prostate enlargement [13], colon carcinogenesis in rats [14], and protecting against hyperglycemia [15], hypertension, [16] and hypercholesterolemia [17]. Several studies have demonstrated the beneficial effects of MC against diabetes by regulating GLP-1, which is crucial in stimulating insulin gene transcription [18]. In addition, chamomile oil significantly decreased osteoarthritis [19], and may also help treat lung cancer [20]. Mondal et al. (2022) found that MK modulated various cellular programs and signaled cascades to intervene as an antioxidant in normal cells and as a pro-oxidant in lung carcinoma cells [21], protecting against liver damage caused by TPA [22]. Moreover, previous studies on the MK leaf fraction have observed its efficacy in treating hyperglycemia [23]. Although UD, MC, and MK have been used in several in vivo and in vitro experiments, there is still little evidence supporting of the therapeutic effect of these three herbs on cellular and neurological complications.

Considering the general protective and cognitive effects of UD, MC, and MK that have been reported in the literature. This study aimed to determine the bioactive compounds of these botanicals (UD, MC, and MK) through biochemical and analytical (UV-Vis, FTIR, and HPLC) methods. Furthermore, we attempted to investigate the cellular and neurological toxicities in a nontarget in vivo model of *D. melanogaster* through a widely used neurotoxic natural pesticide, rotenone (ROT). In addition, we also investigated its organismal effect on *Drosophila*, as it has been demonstrated to be a model framework of neurodegenerative diseases, particularly Parkinson’s disease.

## 2. Materials and Methods

### 2.1. Chemicals and Reagents

Ellman’s reagent (DTNB), 2,2′-azinobis (3-ethylbenzothiazoline-6-sulfonic acid) (ABTS), gallic acid (C_7_H_6_O_5_), 1,-1-diphenyl-2-picrylhydrazyl (DPPH), ascorbic acid (C_6_H_8_O_6_), sodium carbonate (Na_2_CO_3_), trichloroacetic acid (C_2_HCl_3_O_2_), sulfuric acid (H_2_SO_4_), ferric chloride (FeCl_3_), ethanol, gallic acid (C_7_H_6_O_5_), and aluminum chloride hexahydrate (AlCl_3_ 6H_2_O) were obtained from Hi-Media (Mumbai, India). The solvent utilized for HPLC analysis was of HPLC grade, whereas all other organic solvents and chemicals were of analytical grade. Rutin, quercetin, rotenone, and acetylcholinesterase were obtained from Sigma (Roedermark, Germany). Millipore grade water was used. Calorimetric analysis was performed using a Shimadzu UV-1601 spectrophotometer (Tokyo, Japan).

### 2.2. Plant Materials Used in the Study

The young leaves of *Urtica dioica* (UD) were collected from the local kitchen gardens and apple orchards of Sopore (Sopore, India) before the plants started developing seeds. *Matricaria chamomilla* (MC) flowers were procured from the Mediaroma Agro Producer Company Limited Kaskanj (Kaskanj, India), and young leaves of *Murraya koenigii* (MK) were obtained from the garden maintained by Lovely Professional University (Phagwara, India). The identification and authentication of the medicinal plants were confirmed by a taxonomist from the Plant Sciences Division of the CSIR-Indian Institute of Integrative Medicine (IIIM) (Jammu, India).

#### 2.2.1. Drying, Processing, and Extraction of Samples

The collected plant leaves and flowers were washed under tap water and dried at room temperature for a week. After drying, all dry plant components were crushed to a fine powder with a mechanical grinder and then sieved through a 40-micron sieve to obtain fine particles. A 10% aqueous extract was prepared using five grams (±0.05) of the powdered sample mixed with 50 mL deionized water and steeping at 95–100 °C for 10–15 min. Then, the extracts were filtered by using Whatman No. 1 filter paper, and a 10% ethanolic extract was prepared using Soxhlet. Twenty grams (±0.05) of powdered plant samples were inserted into a Whatman 25 × 100 mm celluloid thimble by adding 200 mL of ethanol as a solvent at boiling temperature (70 °C). A dark green extract was obtained from UD and MK leaves, whereas the extract obtained from MC flowers was a pale yellow. The extract was then evaporated using a vacuum rotatory evaporator at 70 °C or less to remove the solvents. The crude extracts were weighed and stored at 4 °C in an airtight dark box for future assessment [24].

#### 2.2.2. Determination of Plant Yield

The percentage yields of UD, MC, and MK samples were calculated using the following formula [25]:

W_2_ − W_1_ × 100 (1)

where W_2_ signifies the weight of the extract including the container, W_1_ signifies the weight of the container itself, and W_0_ signifies the weight of the initial dried sample.

#### 2.2.3. Free Radical Scavenging Activity Using the DPPH Radical Assay

Antioxidant potential was measured using the 1,-1-diphenyl-2-picrylhydrazyl (DPPH) test [26]. A freshly prepared solution of DPPH (0.011 gm) was taken in 50 mL methanol for spectrophotometric measurements. The DPPH solution was further diluted with methanol, and the optical density (OD) was set between 0.8–1. Different concentrations of plant fractions were added to every 2 mL of DPPH mixture. Absorbance was measured at 517 nm using a Shimadzu UV-1601 spectrophotometer (Tokyo, Japan) after 30 min of incubation. Methanol was used as a blank, and DPPH was used as a control. Triplicate experiments were performed. Using the following equation, the radical scavenging activity was calculated as percent inhibition (1%) of the DPPH radical:

DPPH inhibition (%) = [(A_control_ − A_sample_)/A_control_] × 100 (2)

where A_sample_ represents absorbance of the plant extract sample and A_control_ represents absorbance of the DPPH solution as a control.

#### 2.2.4. ABTS Radical Cation Decolorization Assay

The 2,2′-azinobis (3-ethylbenzothiazoline-6-sulfonic acid) radical cation decolorization analyte was also used to assess the efficacy of plant fractions to scavenge free radicals, which is based on the reduction of ABTS radicals by antioxidants in plant extracts [27]. The radical cation formed when the ABTS stock solution (0.036 g in 10 mL methanol) was mixed with potassium persulfate (0.057 g in 10 mL methanol) at a 1:1 ratio. Then, the mixture was incubated in darkness for 16 h at ambient temperature. To attain an optical density (OD) of 0.8–1, the ABTS solution was further diluted with methanol. Every 2 mL of ABTS solution had extracts of various concentrations added to it. All samples were measured at 745 nm after 30 min of incubation. The percentage of scavenging activity was determined using the following equation:

ABTS inhibition (%) = [(A_control_ − A_sample_)/A_control_] × 100 (3)

where A_sample_ signifies absorbance of the plant extract sample and A_control_ signifies absorbance of the ABTS solution as a control.

#### 2.2.5. EC_50_ (Dose-Response Relationship)

EC_50_ is the half-maximal effective concentration of an antibody, drug, or toxicant that elicits a reaction halfway between the baseline and maximum, following a specific duration of exposure. Using CompuSyn software, data analysis for free radical scavenging activity and dose-response studies were performed to assess the potency of the selected herbs. A lower EC_50_ indicates greater radical scavenging activity [28]. SPSS software was used for statistical analysis.

#### 2.2.6. Determination of Total Phenolic Content (TPC)

The TPC of the plant fractions was measured by using Folin–Ciocalteu’s colorimetric method [29]. Each plant fraction was coupled with 2.5 mL of the FC reagent (1:10 *v*/*v*) and vortexed. After 5 min, 2 ml of Na₂CO₃ (7.5%) were incorporated. Then, the solution was placed for approximately 90 min at room temperature before taking the OD at 760 nm by using a UV-Vis spectrophotometer. The results are given in mg GAE/g of dry weight. Triplicates of each sample were analyzed.

(4) $$C=\frac{c\times V}{m}$$

where ‘C’ indicates the total phenolic component content in (mg g^−1^) plant extract in GAE, ‘c’ indicates the gallic acid concentration (mg mL^−1^), ‘V’ indicates the volume of extract in microliters (µL), and ‘m’ indicates the weight of crude plant extract in grams.

The correlation coefficient (R^2^) value was determined using the mean of three absorbance determinations for each concentration. The equation is shown below:

Y= mx + c(5)

where ‘Y’ signifies extract absorbance, ‘m’ signifies the slope of the calibration curve, ‘x’ signifies extract concentration, and ‘c’ is the intercept. Concentrations of extracts were calculated using this regression equation. The phenolic content was estimated using the calculated value for each extract concentration.

#### 2.2.7. Determination of Total Flavonoid Content (TFC)

Total flavonoids were quantified using the aluminum chloride colorimetric technique [30]. The plant extracts were combined with methanol (1.5 mL), 100 µL of (10%) aluminum chloride followed by 0.1 mL of potassium acetate (1 M), and finally 2.8 mL of deionized water. The reaction mixture was placed for 40 min at ambient temperature, and the absorbance of the solution was obtained at 415 nm. Quercetin was used to create a calibration curve. The total flavonoid content was calculated in terms of quercetin equivalents (mg QE/g dry weight). Triplicate readings were taken for each plant sample.

(6) $$C=\frac{c\times V}{m}$$

where ‘C’ is the total phenolic content in mg g^−1^ plant extract in E, “c” reflects the quantity of quercetin determined by the calibration graph (mg/mL), ‘V’ shows the volume of plant extract in µL and ‘m’ is the weight of crude plant extract in grams. The absorbance of each concentration of the extract was measured using the procedure described above. Then, using the calculations above, the total flavonoid content was determined.

#### 2.2.8. Preliminary Qualitative Screening Analysis of Plant Extracts

Bioactive compounds such as polyphenols, reducing sugars, alkaloids, terpenoids, glycosides, flavonoids, saponins, and amino acids are mostly responsible for curative capabilities such as menstruation problems, muscular spasms, anemia, ulcers, hemorrhoids, inflammation, and wound healing [31]. The presence of phytochemicals is determined using conventional qualitative test methods, which include the following:

(a) *Detection of phenols (ferric chloride test):* An amount of 2 mL of plant extract was combined in a test tube with 2 mL of 5% ferric chloride aqueous solution. The presence of phenols was indicated by a deep blue-green solution [32].
(b) *Detection of flavonoids (Alkaline reagent test):* A few drops of NaOH (20%) solution were added to 2 mL of plant extract, which displayed a yellowish red color within a second and turned transparent with the addition of diluted HCl, displaying a positive result [33].
(c) *Detection of alkaloids (Wagner’s test):* To 4 mL of plant extract, 3 drops of Wagner’s reagent were added. The appearance of a reddish-brown precipitate indicated a positive outcome [33].
(d) *Detection of tannins (FeCl3 solution test):* An alcoholic ferric chloride (10%) solution was added to 2 mL of plant extract. The appearance of the blue/green color suggested a positive outcome [33].
(e) *Detection of carbohydrates (Molisch’s test):* An amount of 3 mL of extract and 3 mL of H_2_SO_4_ were placed in a test tube; a few drops of Molisch’s reagent were added (conc.). Allowing it to stand 3 min, the appearance of a red/dull violet tone at the interphase of the two layers showed a successful outcome [32].
(f) *Detection of saponins (saponin foam test):* Five milliliters of distilled water was combined with 500 µL of plant fractions. The presence of saponins is indicated by foaming (formation of creamy tiny bubbles) [34].
(g) *Detection of terpenoids (Salkowski test):* An amount of 2 mL of extract and a few drops of conc. H_2_SO_4_ was mixed with 1 mL of chloroform. The appearance of a reddish-brown precipitate indicated a positive outcome [32].
(h) *Detection of steroids (Liebermann-Burchard test):* An amount of 3 mL of acetic anhydride was mixed with 5 mL of plant extract. Then, 2 mL of H_2_SO_4_ was added to it. The presence of steroids was indicated by a shift in color from violet to bluish green [34].
(i) *Detection of glycosides (Kellar-Kiliani test):* An amount of 1 mL of glacial acetic acid was mixed with 2 mL of plant extract. Then, 1 mL of FeCl_3_ and 1 mL of (conc.) H_2_SO_4_ was mixed into it. The appearance of glycosides was confirmed by the solution’s greenish-blue color [34].

#### 2.2.9. UV-Visible Spectroscopic Analysis

The ultraviolet spectral data were obtained by a Shimadzu UV-1601 spectrophotometer (Tokyo, Japan). UV-Vis spectroscopy is concerned with the absorption of radiation in the ultraviolet and visible spectra and mostly used for quantitative analysis [35]. This radiation permits electrons in atoms or molecules to shift from lower to higher energy levels. The level of radiation absorbed is proportional to the number of molecules in the solution under specified conditions. Spectral data were used to demonstrate a link between absorption concentration and intensity. Quality control might thus be evaluated with a UV-Vis spectrometer without the need for expensive markers by establishing a library of spectrum data from actual raw samples [36]. Extracts of UD, MC, and MK were used for UV-Visible analysis. The samples were tested with a spectral range of 200–800 nm at 1 nm intervals at room temperature.

#### 2.2.10. FT-IR Spectroscopic Analysis

Fourier transform infrared spectrophotometry (FTIR) is one of the most potent instruments for detecting types of chemical bonds, molecular structures, and functional groups in substances. The absorbed wavelength of light is indicative of the chemical bond, as shown in the annotated spectrum. The chemical bonds of a molecule can be identified by interpreting the infrared absorption spectrum [37].

Ethanolic extracts of UD, MC, and MK were used for FTIR analysis. To make translucent sample discs, 10 mg of the crude plant extract sample was enclosed in 100 mg of KBr pellet. Then, the crude extract of each plant material was subjected to FT-IR spectroscopy (Perkin-Elmer Spectrum 2 with ATR and Pellet accessories). The samples were tested in the infrared band with a spectral range of 400–4000 cm^−1^ and a resolution of ±4 cm^−1^ at room temperature.

#### 2.2.11. HPLC Chromatographic Analysis

A Shimadzu Prominence I LC2030 Plus HPLC system (Kyoto, Japan) equipped with a Shimadzu LC 2030 UV-Vis detector was used to separate natural compounds from crude extracts of UD, MC, and MK. The standard external technique was used to perform HPLC analysis under isocratic conditions. Before running in the column, the mobile phase was degassed and filtered through a membrane using methanol and 0.5% acetic acid in water (90:10 *v*/*v*). Column C-18 (4.6 × 250 mm) with a 5 µm particle size was used and maintained at 25 °C temperature. Each injection volume was prepared at 20 µL and then injected into the HPLC. Samples were filtered using a 0.45 mm membrane filter (Millipore) before being put in vials, employing a 1.0 mL/min flow rate.

Spectral information was analyzed in the 200–400 nm region, and chromatograms were detected at a wavelength of 280 nm. Based on previous findings, the quantitative quantification of each bioactive component contained in the plant extracts was determined [38,39,40]. Peak identification was carried out by comparing the retention times of specific standards with those of the extract. The retention times of specific standards were compared with those of the extract to identify the peak. 

### 2.3. Fly Strain and Maintenance of Culture

A wild-type *Drosophila melanogaster* strain (Oregon R^+^) that was kindly gifted by Dr. Anurag Sharma, Senior Assistant Professor, NITTE (Deemed to be University), Mangalore, India, and maintained on a standard *Drosophila* diet (containing maize powder, agar-agar, sugar, yeast, sodium benzoate, and propionic acid) was used for rearing. The flies were retained in a 12-h dark/light cycle at 24 ± 1 °C and 65–70% humidity levels. [41].

#### 2.3.1. Plant Concentration and Rotenone Exposure

To determine whether the treatment has any impact on the survival of the flies during the experimental period, preliminary studies were conducted with small numbers of flies, testing several concentrations (0.01, 0.025, 0.05, and 0.1%) of UD, MC, and MK. However, only one concentration, i.e., 0.1% per unit of the medium, was selected as the optimum concentration from the conclusive studies. Nevertheless, to evaluate the cellular and neurological protective properties of UD, MC, and MK, the concentration of rotenone (500 µM) used was selected based on our findings in *Drosophila* and those of other published studies [42,43,44].

#### 2.3.2. Treatment Schedule

For the experimental setup, flies/third instar larvae of *Drosophila* were divided into six groups. There were two groups of controls: Group I was fed the larval standard *Drosophila* food as a control, whereas Group II was fed food mixed with 0.1% DMSO as a vehicle control. Group III represented ROT (500 µM) treatment alone; Group IV comprised ROT with UD extract (0.1%); Group Ⅴ consisted of ROT and MC extract (0.1%); Group VI was ROT cotreated with MK extract (0.1%). Larvae were permitted to feed either normally or with food that had been exposed to ROT or ROT+ fractions for 24 and 48 h. The flies were exposed for 120 h (5 days) and were assessed for jumping and climbing. We determined the modulatory effect of UD, MC, and MK fractions on rotenone-induced lethality, locomotor dysfunctions, inhibition of acetylcholinesterase, cellular toxicity, and proteotoxicity.

#### 2.3.3. Trypan Blue Dye Exclusion Assay

Dye exclusion was employed as described by Krebs and Feder (1997) with slight modifications [45]. This is a simple and quick method that distinguishes living and nonliving cells in tissue. It is used to detect cell death in the whole larvae and larval gut. At the end point of treatment, 5–10 larvae were thrice washed with 0.1 M phosphate buffer saline (pH 7.4), then whole or dissected midguts of larvae were incubated in trypan blue solution (0.2 mg/mL in 50.0 mM PBS, pH 7.4) for 15 min followed by three washes with 0.1 M phosphate buffer saline (pH 7.4). The larvae were analyzed by using a stereomicroscope; the images were acquired for trypan blue scoring, and were analyzed thoroughly.

#### 2.3.4. Homogenate Preparation

To obtain 10% homogenate/cytosol, the midgut of the third instar larvae was dissected from control (normal/untreated), DMSO, ROT, and ROT with phyto extract groups and homogenized in ice-cold 0.1 M phosphate buffer at a pH of 7.4, containing 0.15 M KCl. Following homogenization, the samples underwent centrifugation at 10,000× *g* at 4 °C for 15 min. After that, a nylon mesh sieve with a pore size of 10 mm was used to filter the supernatant, which was then used for various assays. [46].

#### 2.3.5. Protein Estimation

The method of Lowry et al. (1951) was used to measure the protein concentrations in the whole-body homogenates using bovine serum albumin as the standard [41].

#### 2.3.6. Acetylcholinesterase (AChE) Enzymatic Assay

AChE activity was measured as previously mentioned [47]. Briefly, the reaction was started by adding acetylthiocholine iodide (78 mM) to 0.1 M phosphate buffer with a pH of 8.0, which also contained 5,5’-dithio-bis-(2-nitrobenzoic acid) (DTNB 10 mM) and sample (cytosolic) 0.01 mg protein. The change in absorbance was observed at 412 nm for three minutes. The amount of substrate hydrolyzed/min/mg protein was used to express enzyme activity.

#### 2.3.7. Measurement of Locomotor Deficits: Climbing Assay

The climbing assay was performed with some modifications, as previously described [48]. A vertical plastic tube measuring 18 cm in length and 2 cm in diameter was filled with twenty adult flies. During a 30 s time frame, flies followed tapping sounds until they reached the bottom of the vials; flies were scored if they crossed the 15 cm line. The average percentage of flies that cross the 15 cm line is represented by the climbing scores. The average number of flies above and below 15 cm (*n*_top_ and *n*_bot_), expressed as a percentage of the total number of flies, determines the scores (*n*_tot_). The results are displayed in ±SD of the results from three independent observations. A performance index (PI) was calculated for each experiment and was given as follows: 1/2[(*n*_tot_ + *n*_top_ − *n*_bot_)/*n*_tot_].

#### 2.3.8. Jumping Assay

The jumping activity was performed to examine neuromuscular activity [49]. The speed of locomotor activity seems to have an impact on the threshold for the jumping reaction. One at a time, newly emerged flies were put into a labelled vial 1–10 cm, and the height the fly jumped from the bottom of the vial was recorded. The jumping activity was determined to be the mean number of jumps across five replicates. Five replicates of each group of 100 flies each were used.

### 2.4. Statistical Analysis

To analyze the UV-Vis, FT-IR, and HPLC profiles, professional assessment software was used (Digitized Quantitative Evaluation System of Herbal Medicine Chromatographic Fingerprints 4.0). EC_50_ analysis was performed using CompuSyn software (version 1.0). Chemometric data were expressed as the mean ± SEM, and using SPSS software (version 18), a two-way ANOVA and Tukey’s test were used to compare n = 3 for significant differences. Significance was ascribed to a *p* value < 0.05.

## 3. Results

The results of the assays and analyses used to determine the feasibility of UD, MC, and MK extracts against rotenone-induced lethality, locomotor dysfunctions, inhibition of acetylcholinesterase, cellular toxicity, and proteotoxicity are presented in the subsections below.

### 3.1. Analytical Assays

#### 3.1.1. Percentage Yield of Bioactive Compounds

The results showed that MK leaf extraction yield was higher than that of UD and MC when distilled water was used as the extracting solvent (Table 1). The yield of ethanolic extraction was again higher in MK, followed by MC and UD. The findings also revealed that variation in the yield depends on the extraction solvent used. The variability in extract quantities from the plant materials used in this study could be due to the varying accessibility of extractable components caused by plant position.

#### 3.1.2. Antioxidant Potential of UD, MC, and MK

**Figure 1:**
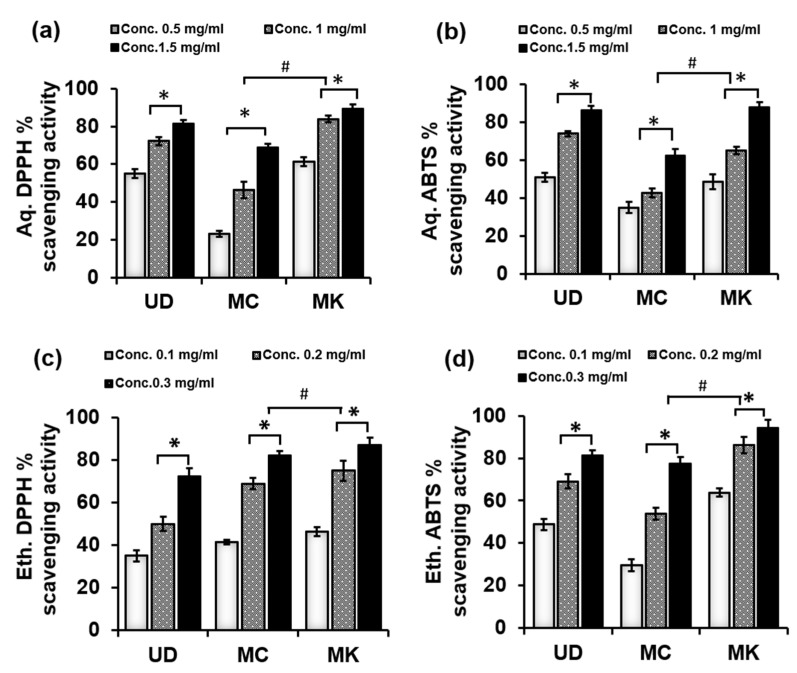
The effect of different concentrations of *U. dioica* (UD), *M. chamomilla* (MC), and *M. koenigii* (MK) in (**a**,**b**) aqueous and (**c**,**d**) ethanolic extractions on the (**a**,**c**) DPPH and (**b**,**d**) ABTS free radical scavenging assay. Data represent mean ± SD for n = 3. Statistically significance ascribed as * *p* < 0.05 (intragroup) and ^#^ *p* < 0.05 (intergroup) compared with 0.5 mg/mL and 0.1 mg/mL of the respective groups.

**Figure 2:**
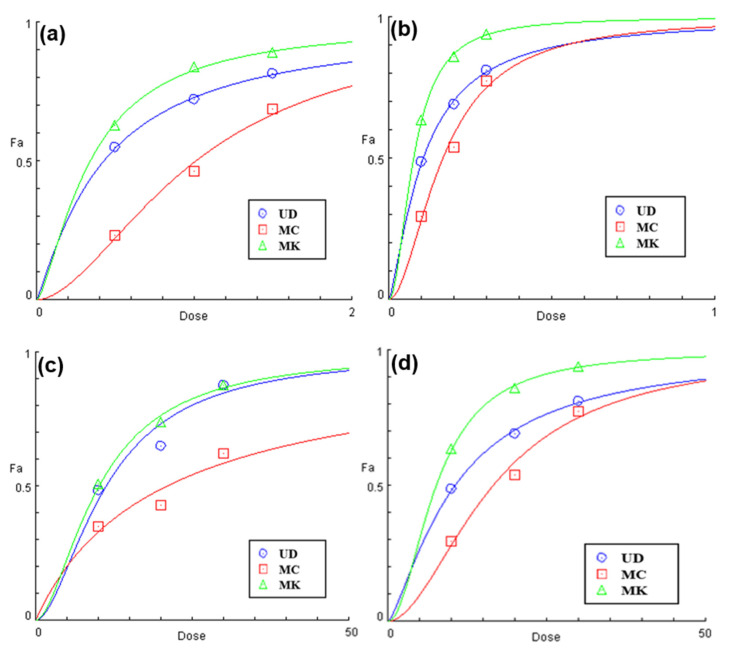
Dose-response profiles of the estimated EC_50_ (mg/mL) of (**a**,**c**) aqueous and (**b**,**d**) ethanolic extracts of *U. dioica* (UD), *M. chamomilla* (MC), and *M. koenigii* (MK) on the (**a**,**b**) DPPH and (**c**,**d**) ABTS assays.

DPPH is widely used to evaluate the antioxidant and antiradical potential of plant fractions. The ability of antioxidants to scavenge DPPH radicals is assumed to be attributed to their hydrogen donating capabilities. The antioxidant potential of both aqueous and ethanolic extracts of UD, MC, and MK was measured using a free radical DPPH assay. As shown in Figure 1a and Figure 2c, MK had the highest DPPH radical scavenging activity in both aqueous and ethanolic extracts, followed by UD and MC. Compared with aqueous extracts, ethanolic extracts of all three plants had the strongest scavenging efficacy.

ABTS is an unstable colored free radical that is used to investigate the antioxidant properties of both hydrophobic and hydrophilic antioxidants found in food extracts. The ABTS radical scavenging activity of the different extracts with different extraction solvents (aqueous and ethanol, Figure 1b and Figure 2d) was found to be higher in MK, followed by UD and MC. These results suggest that MK has a higher efficacy in scavenging free radicals along with greater antiradical and antioxidant activity.

#### 3.1.3. EC_50_ Prediction Using Statistical Models

EC_50_ is a significant parameter for assessing antioxidant activity and can be used to compare the antioxidant capacities of different materials. The EC_50_ can be calculated, using various models, by interpolating data from a suitable curve or performing a nonlinear regression of the data using different components. The leaves of MK were found to have a higher antioxidant capacity due to its greater total phenolic and flavonoid content, with EC_50_ values of 0.33 and 0.10 mg/mL for aqueous and ethanolic extractions on DPPH scavenging and 0.51 and 0.07 mg/mL for aqueous and ethanolic extractions on ABTS radicals, respectively (Figure 2; Table 2).

#### 3.1.4. Phenolic and Flavonoid Potential of UD, MC, and MK

**Figure 3:**
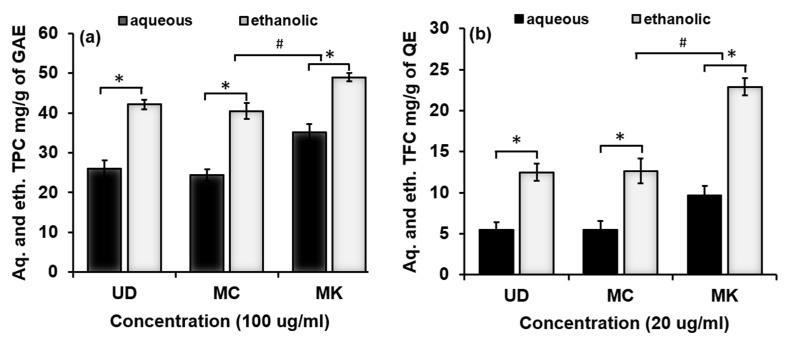
Total (**a**) phenolic and (**b**) flavonoid contents of aqueous and ethanolic fractions of *U. dioica*, *M. chamomilla*, and *M. koenigii*. Data are shown as the mean ± SD for n = 3. Statistical significance is ascribed as * *p* < 0.05 (intragroup) and ^#^ *p* < 0.05 (intergroup) of the respective groups.

Individual aqueous and ethanolic extracts of UD, MC, and MK were quantified for total phenolic and flavonoid content. The total phenolic content (TPC) was calculated based on the gallic acid standard curve. In terms of aqueous extracts, MK (35.14 mg (GAE)/g) was found to have the highest TPC, followed by UD (26.08 mg (GAE)/g), then MC (24.01 mg (GAE)/g). The trend was the same in the case of ethanolic extracts: MK (48.93 mg (GAE)/g) was followed by UD (42.93 mg (GAE)/g) and was lowest in MC (40.5 mg (GAE)/g), as shown in Figure 3 and Table 3. The total flavonoid content (TFC) was calculated based on the quercetin standard curve. It was highest in MK leaves (9.64 mg (QE)/g), followed by MC flowers (5.46 mg (QE)/g), and lowest in UD leaves (5.45 mg (QE)/g) in the case of aqueous extracts, and the trend was the same for ethanolic extracts: MK (22.88 mg (GAE)/g), MC (12.64 mg (GAE)/g), and UD (12.48 mg (GAE)/g), as shown in Figure 3 and Table 3. The total amounts of phenolic and flavonoid content in the aqueous fractions of UD, MC, and MK were lower than in the ethanolic extracts. This may have been due to the aqueous solvent and extraction process employed. It has been shown that the solvent employed for extraction may be to blame for the quantity of phenolic content in the extract [24]. MK had the highest TPC and TFC levels compared with UD and MC in both aqueous and ethanolic extracts. The presence of hydroxyl groups in phenols allows them to scavenge free radicals, proving that they are the most essential phytochemicals.

#### 3.1.5. Preliminary Qualitative Screening Analysis of Plant Extracts

Phytochemicals are chemical molecules produced by plants due to their normal metabolic activities. These substances are referred to as secondary metabolites. There is still limited understanding of the benefits of plants because of a lack of raw data and experiments following proper scientific standards. Phytoconstituents such as amino acids, polyphenols, reducing sugars, alkaloids, terpenoids, glycosides, carbohydrates, and saponins are mostly responsible for plant curative capabilities, such as menstruation problems, muscular spasms, anemia, ulcers, hemorrhoids, inflammation, and wound healing. To confirm the existence of phytoconstituent substances, a phytochemical screening study was performed on the crude extracts of UD, MC, and MK, along with appropriate chemical tests. The presence of bioactive phytochemical substances such as phenols, flavonoids, alkaloids, tannins, carbohydrates, saponins, terpenoids, steroids, and glycosides are represented in Table 4**.**

#### 3.1.6. UV-Visible Analysis

**Figure 4:**
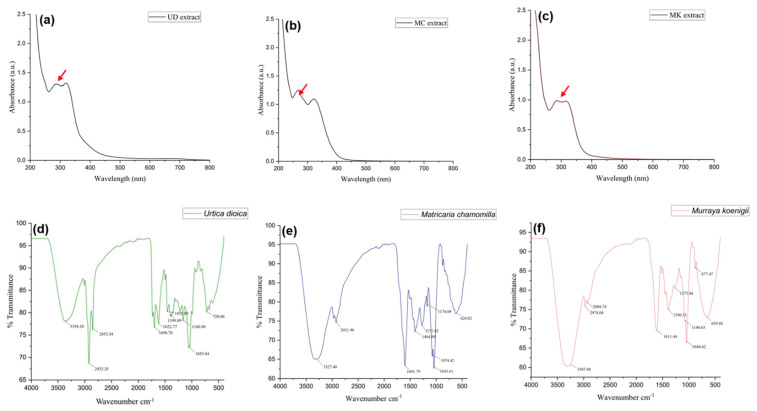
UV-Visible spectra of (**a**) UD, (**b**) MC, and (**c**) MK fractions. Red arrows represent the peak of extract (conjugated or chemical bonds). The FT-IR absorption spectrum of (**d**) UD, (**e**) MC, and (**f**) MK with a scan range of 400–4000 cm^−1^.

Spectral data were observed in the range of 200–800 nm at intervals of 1 nm and showed maximum absorption at 277 and 321 nm in the case of UD, 266 and 323 nm for MC, and 286 and 316 nm for MK (Figure 4). With increasing concentration, absorption increased. This technique is a quantitative analysis of UD, MC, and MK and their absorption of radiation in the ultraviolet and visible spectra. This radiation enables electrons in atoms or molecules to shift from lower to higher energy levels. Under regulated conditions, the amount of radiation absorbed is proportional to the intensity of chemicals in the plant extracts. Spectral analysis showed peaks in crude extracts of UD, MC, and MK, indicating the presence of a variety of chemicals, particularly providing information about unsaturated bonds in conjugated or aromatic components.

#### 3.1.7. Fourier Transform Infrared Spectrophotometer (FTIR)

The functional groups of bioactive components present in plant extracts of UD, MC, and MK were identified using the FT-IR spectra depending on peak values in the IR radiation area. When the extract was run through FTIR, the functional groups of the constituents were segregated based on the ratio of their peaks. The existence of alkanes, amino acids, aldehydes, phenols, secondary alcohols, aromatic amines, ketones, and halogen compounds was verified by FTIR analysis (Figure 4).

In the ethanolic extracts of UD, MC, and MK, the band between 3500 and 3200 cm^−1^ was assigned to an O–H stretching, indicating the presence of hydroxyl and phenolic groups. These groups are found in cellulose, hemicellulose, and lignin structures and could be related to the hydroxylated compounds (polyphenols) and moisture content of the extract. A minor intensity peak in the region of 2950–2960 cm^−1^ showed an O–H (stretch), suggesting the existence of amide groups and alcohols, as well as C–H vibrations of the CH_3_ group. In the area of 1650–1600 cm^−1^, an acceptable N=O (stretch) vibration was recorded. The weak peaks in this area suggest the existence of C=C (stretch). Absorption in this region may indicate the existence of carbonyl groups. A doublet band was seen in the fingerprint area between 1455 and 1370 cm^−1^, indicating the C-H (bending) vibration of the methyl group (–CH_3_) molecule. Furthermore, a broad peak was found between 1100 and 1000 cm^−1^, suggesting the presence of alcohol groups within the compound structure. This is most likely due to an alkoxy C–O (stretching) vibration. In addition, the UD, MC, and MK fractions showed aromatic C–H bonds between 720 and 620 cm^−1^ in the infrared spectrum (Table 5).

Finally, the results revealed that the chemical structures of UD, MC, and MK are extremely polar (lignin (+)-neoolivil, 3,4-divanillyltetrahydrofuran, isolariciresinol, (−)-secoisolariciresinol, and pinoresinol), which stimulates the proliferation of human lymphocytes and has anti-inflammatory effects, as evidenced by the presence of wide peaks within the spectra.

#### 3.1.8. High-Performance Liquid Chromatography (HPLC)

**Figure 5:**
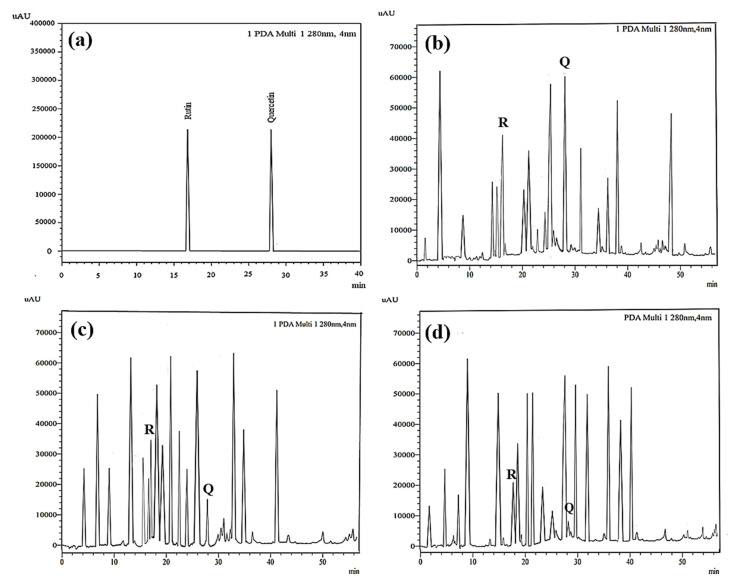
HPLC profiles acquired at 280 nm of (**a**) standard rutin and quercetin and ethanolic extracts obtained from (**b**) *U. dioica* leaves, (**c**) *M. chamomilla* flowers, and (**d**) *M. koenigii* leaves, showing different bioactive compounds. R stands for rutin, and Q stands for quercetin.

The external standard technique was used to perform HPLC experiments under isocratic conditions. Ethanolic extracts of UD, MC, and MK were analyzed directly using the total extracts without any manipulation. The retention time of the chromatographic peaks of plant extracts was compared with reference standards (rutin and quercetin), and DAD spectra (200–400 nm) of existing literature were analyzed (Figure 5). Our findings revealed the presence of 24 compounds (Table 6) in the ethanolic extracts of UD, MC, and MK, including quercetin, coumaric acid, chlorogenic acid, gallic acid, apigenin, myricetin, ferulic acid, fumaric acid, rutin, isorhamnetin, kaempferol, etc.

In the ethanolic extract of UD, three classes of phenols were characterized: anthocyanin compounds (rosinidin 3-*O*-rutinoside; peonidin 3-*O*-rutinoside; and peonidin 3-O-(6′-O-coumaroyl glucoside), hydroxycinnamic acid derivatives (p-coumaric acid; chlorogenic acid; caffeoylquinic acid; and 2-O-caffeoylmalic acid), and flavonoids (rutin; isorhamnetin 3-O-rutinoside; quercetin; p-coumaroyl glucoside; kaempferol 3-O-rutinoside; kaempferol 3-O-and glucoside; and quercetin 3-O-glucoside).

The polyphenolic compounds found in the ethanolic fractions of MC flowers were identified as essential constituents, such as quercetin (quercetin-7-O-β-glucoside; quercetin-3-O-β-rutinoside; and quercetin-3-O-β-galactoside), apigenin (apigenin-7-O-7-glucoside; apigenin-7-O-apiosyl-glucoside; and apigenin-7-O-glucosyl-6’-acetate), luteolin (luteolin-7-O-β-glucoside; luteolin-4’-O-7-β-glucoside; and luteolin-7-O-β-rutinoside), isorhamnetin (isorhamnetin-7-O-β-glucoside), patuletin (patuletin-7-O-β-glucoside), eupatoletin, astragalin, chrysosplenol, and spinacetin. The MK ethanolic fraction was examined by HPLC-DAD, which permitted the identification of important components such as chlorogenic acid, quercitrin, citric acid, piperine, 7 p-coumaric acid, hesperidin, rutin, gallic acid, β-terpineol, ferulic acid, catechin, naringenin, D-α-pinene, di-α-phellandrene, dipentene, D-sabinene, caryophyllene, nicotinic acid, koenigine-quinone A, and koenigine-quinone B. All these secondary metabolites have been shown to have cerebrovascular protective, neuroprotective, and cardiovascular protective properties. In addition, it also acts as an anti-carcinogenic, anti-tumor, anti-inflammatory, antimicrobial, antiviral, and antibacterial agent and protects against oxidative stress-related diseases.

### 3.2. Cellular Assays

#### Cytotoxicity of Rotenone and Amelioration of Cytotoxicity through Bioactive Compounds UD, MC, and MK Determined by a Dye Exclusion Test (Trypan Blue) in Whole Larvae and Tissues of ROT-Exposed Organisms

**Figure 6:**
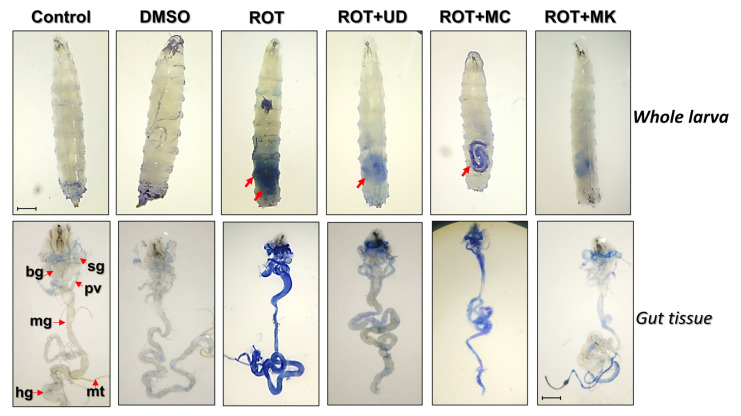
Dye exclusion test through trypan blue staining in third instar larvae exposed to rotenone and cotreated with UD, MC, and MK, as shown in the upper panels. The lower panels show dissected third instar larvae stained with trypan blue. Seventy-two hour (±2 h) old larvae (early third instar) of D. melanogaster (Oregon R^+^) were exposed to ROT 500 µM alone or in combination with UD, MC, and MK for 48 h. Arrows of the upper panel show cytotoxicities in the whole larvae through trypan blue staining. Note: bg= brain ganglia, sg= salivary glands, pv= proventriculus, mg= midgut, mt= malpighian tubules, and hg = hind gut. The bar represents 100 μm. ROT= rotenone; UD = *Urtica dioica*, MC = *Matricaria chamomilla* and MK = *Murraya koenigii*.

To determine if exposure to ROT causes any tissue damage, we analyzed trypan blue staining in whole larvae and tissues of D. melanogaster (Figure 6). Of the larvae exposed to ROT, 95% showed blue staining in the whole larvae and their tissues (brain ganglia, salivary gland, midgut, and gastric caeca). ROT coexposed with MK exhibited significantly less blue staining than in the ROT + UD and ROT + MC groups in the whole larvae and the abovementioned tissues, respectively.

### 3.3. Biochemical Assays

#### 3.3.1. Decreased Protein Content in *D. melanogaster* Treated with Rotenone after 24 and 48 h

**Figure 7:**
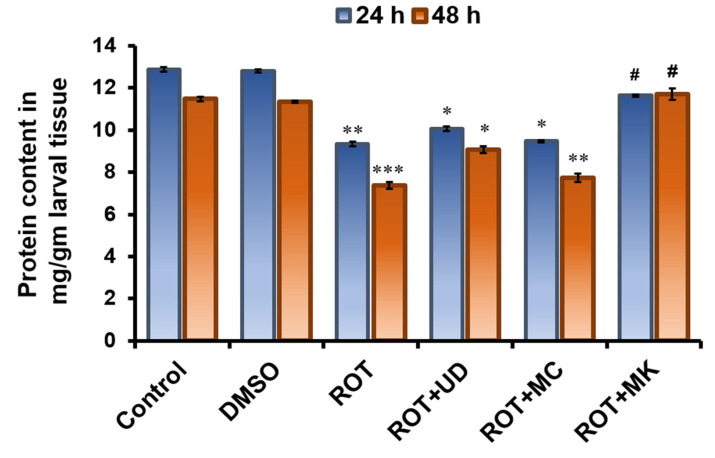
Total protein content in third instar larvae of *D. melanogaster* (Oregon R^+^) exposed to 500 µM rotenone for 24 and 48 h. Data represent the mean ± SD of three identical experiments made in three replicates. Significance is ascribed as * *p* < 0.05, ** *p* < 0.01, *** *p* < 0.001 vs. control or DMSO control. ^#^ = significance at * *p* < 0.05 as compared with 500 µM rotenone. UD = *Urtica dioica*, MC = *Matricaria chamomilla* and MK = *Murraya koenigii*.

Third instar larvae of *D. melanogaster* exposed to 500 µM ROT exhibited a significant reduction (*p* < 0.05) in the total protein content of their tissues. After 24 h, the protein content in the larvae was reduced in the ROT group (9.34 ± 0.150 mg/mL) compared with the control group (12.88 ± 0.313 mg/mL). ROT coexposed with MK (11.63 ± 0.225 mg/mL) showed highest protein levels, followed by ROT + UD (10.05 ± 0.381 mg/mL), and ROT + MC (9.46 ± 0.174 mg/mL). After 48 h, in comparison with the control group (11.47 ± 0.328 mg/mL), the ROT treatment decreased the protein content in the larvae (7.37 ± 0.225 mg/mL). Increased protein levels were observed in ROT coexposed with MK (11.71 ± 0.263 mg/mL), followed by ROT + UD (9.07 ± 0.196 mg/mL), and ROT + MC (7.72 ± 0.213 mg/mL) (Figure 7).

#### 3.3.2. Rotenone Inhibits AChE Activity in *D. melanogaster*, and This Effect Is Reversed by Phytoextraction

**Figure 8:**
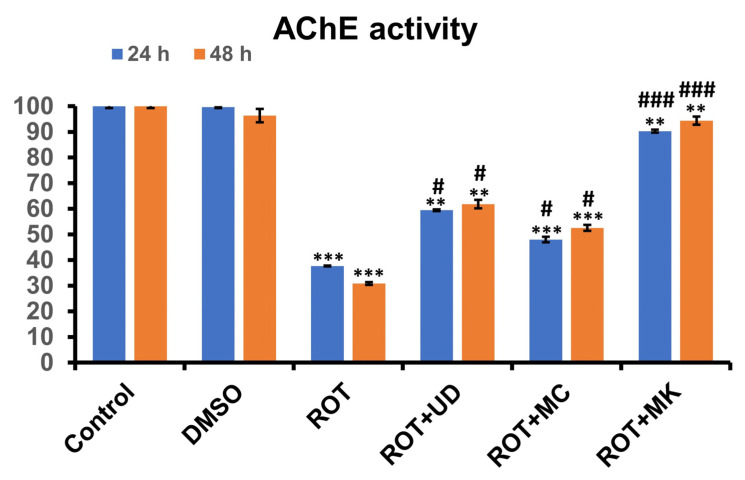
Acetylcholinesterase activity in the third instar larvae of *D. melanogaster* (Oregon R^+^) exposed to 500 µM ROT alone or in combination with UD, MC, and MK for 24 and 48 h. Data represent mean ± SD (n = 3); significance ascribed as ** *p* < 0.01, *** *p* < 0.001 vs. control or DMSO control. ^#^ is ascribed as significance at *p* < 0.05, ^###^ *p* < 0.001 as compared with 500 µM rotenone. UD = *Urtica dioica,* MC = *Matricaria chamomilla* and MK = *Murraya koenigii*.

In this study, it was found that when the larvae were exposed to ROT for 24 h, they exhibited statistically significant (*p* < 0.001) inhibition of AChE activity compared with the control or DMSO, and ~60% reduced AChE levels were observed in this group. When ROT was coexposed with MK, AChE levels were improved, and only 9.7% inhibition was evident compared with the control. These elevated levels of AChE were significant when compared with the ROT-treated groups. The AChE levels in the ROT + UD and ROT + MC groups were also significantly improved (40.5% and 52.0% inhibition, respectively). Maximum inhibition of AChE levels was present in ROT-exposed organisms after 48 h (69.13% compared with control larvae), and the greatest improvement from ROT-induced toxicity was observed in the ROT + MK group (5.63% compared with control groups). Significantly higher AChE levels were also observed in the ROT + UD and ROT + MC groups (38.15 and 47.45% inhibition, respectively) than in the ROT-treated group (Figure 8).

### 3.4. Behavioral Assays

#### 3.4.1. Rotenone Affects Locomotor Behavior in *D. melanogaster*

**Figure 9:**
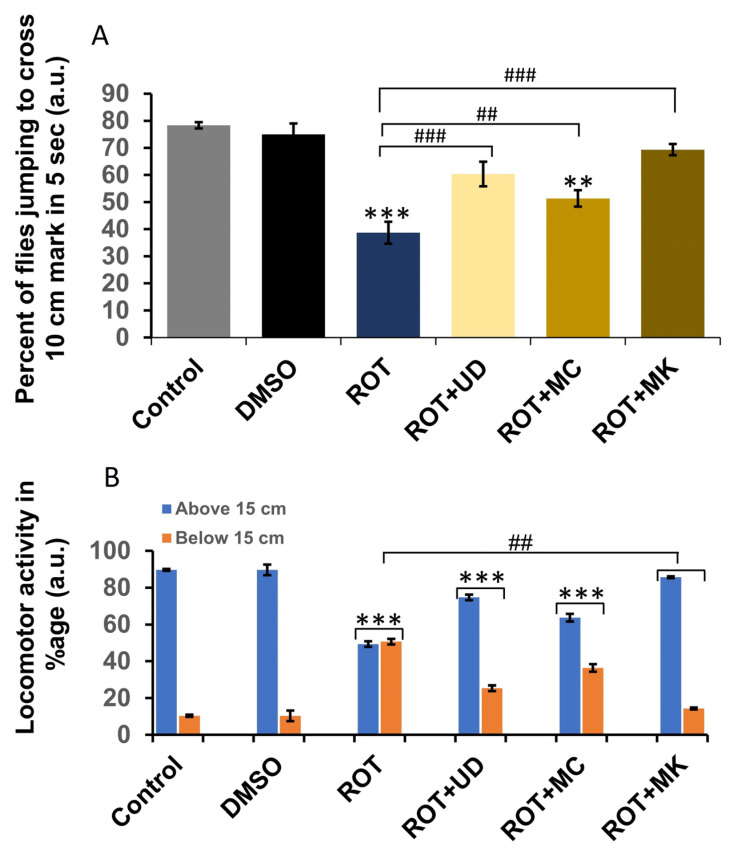
(**A**) Jumping and (**B**) climbing activity of *D. melanogaster* (Oregon R^+^) flies exposed to ROT 500 µM alone or in combination with UD, MC, and MK for 120 h; significance is ascribed as ** *p* < 0.01, *** *p* < 0.001 vs. control or DMSO control. ^##^ ascribed as significance at *p* < 0.01, ^###^ *p* < 0.001 compared with 500 µM rotenone. UD = *Urtica dioica*, MC = *Matricaria chamomilla* and MK = *Murraya koenigii*.

After 30 s, the control and DMSO-treated flies demonstrated maximum climbing ability (only 11% and 12% reduction, respectively). The greatest reduction in climbing ability was observed in ROT-treated *Drosophila* (50.5%), and flies found it difficult to climb the plastic tube walls. The groups receiving ROT + phytoextracts exhibited varying levels of improvement in their climbing skills. All nutraceuticals improved the climbing ability of flies. Among the nutraceutical groups, ROT + MK (15%) exhibited the greatest improvement, followed by ROT + UD (26%), then ROT + MC (37%). To identify any significant differences, the mean ± SEM was compared using an unpaired Student’s *t*-test. Significance was ascribed at *p* < 0.001 (Figure 9A).

#### 3.4.2. Significant Changes in the Jumping Activity of ROT-Exposed Flies

We observed significantly decreased jumping behavior in the flies treated with ROT (62%) compared with the control and DMSO (22 and 25%, respectively). The groups receiving ROT + phytoextracts exhibited varying levels of improvement in their jumping skills. All nutraceuticals improved the jumping ability of flies. ROT + MK (31%) exhibited the greatest improvement, followed by ROT + UD (40%), and ROT + MC (49%). An unpaired Student’s t-test was used to compare the mean ± SEM. Significance was ascribed at *p* < 0.001 (Figure 9A).

## 4. Discussion

The present study demonstrates the protective efficacy of UD, MC, and MK against rotenone-induced cellular, neurological, and organismal toxicity in a nontarget organism, *Drosophila melanogaster*. Unlike synthetic drugs, herbal medicines have a comprehensive structure of chemical elements. As a result, the methods of choice for identifying a ‘botanical medicine’ are primarily designed to obtain a unique fingerprint of certain plants that indicates the existence of quality-defining active chemical elements [50]. It is noteworthy that the Ayurvedic medical system has well-established the benefits of UD, MC, and MK for improving cognition, memory, and learning. To our knowledge, the current study is the first to report the comparative efficacy of UD, MC, and MK against various ROT-induced toxicities in *Drosophila*.

In this study, we examine how different aqueous and ethanolic fractions of UD, MC, and MK react to different radicals. Our results demonstrate that the ABTS activity in MK extracts did not differ significantly from the free radical scavenging potential measured by the DPPH assay because both assays use the same mechanism (single-electron transfer). Among the three extracts, MK displayed the greatest scavenging activity in both the DDPH and ABTS radical scavenging assay, as shown in Figure 1. Previous research has shown that MK has great antioxidant potential [51]. MK leaves had greater antioxidant capacities than UD leaves and MC flowers, owing to greater total phenolic and flavonoid content, with EC_50_ values for aqueous and ethanolic extracts in DPPH scavenging of 0.33 and 0.10 mg/mL, respectively, and EC_50_ values for aqueous and ethanolic extracts in ABTS scavenging of 0.51 and 0.07 mg/mL, respectively. Aqueous extracts of UD, MC, and MK had lower total phenolic and flavonoid concentrations their ethanolic counterparts. This could be due to the aqueous solvent and extraction method used. It was also discovered that the extraction solvent could be at fault for the amount of phenolic content in the extracted samples [52]. In both the aqueous and ethanolic fractions, the TPC and TFC were highest in MK, followed by UD, and MC had the lowest TPC values. This could be due to the extraction solvent used, as ethanolic extraction solvent was previously regarded to be the best for extracting total flavonoids [53]. Overall, MK exhibited the highest antioxidant properties due to the highest TPC, TFC, DPPH, and ABTS, followed by UD and MC. The aromatic leaves of MK have 11–21 pinnate leaflets that are each 2–4 cm (0.80–1.59 in) long and 1–2 cm (0.38–0.80 in) wide. It is known that MK leaves are a major source of phyto-carbazole alkaloids, comprising mahanimbine, koenine, girinimbine, murrayacine, koenidine, mahanine, and 8,8′-biskoenigine, with promising pharmacological activities [51]. Bioactive phytochemical substances of these three herbs, such as phenols, flavonoids, alkaloids, tannins, saponins, terpenoids, steroids, and glycosides, are primarily responsible for curative properties such as menstrual problems, muscular spasms, anemia, ulcers, hemorrhoids, inflammation, and wound healing [54].

UV-Vis spectral analysis was recorded in the range of 200–800 nm with intervals of 1 nm and showed maximum absorption at 277 and 321 nm for UD, 266 and 323 nm for MC, and 286 and 316 nm for MK. Beer’s rule asserts that the amount of light absorbed at a specific frequency is proportional to the sample’s composition absorption coefficient [55]. As a result, all spectral fluctuations caused by spectrophotometer and sample inaccuracies must be rectified before information processing. Peaks in crude extracts indicated the presence of a variety of ingredients or chemicals, particularly unsaturated bonds in conjugated or aromatic components [56].

A Fourier transform infrared spectrometer was used to determine the functional groups of the bioactive components in the plant fractions based on peak intensity in the infrared radiation (IR) region. The specific wavenumbers and intensities were determined in the range of 4000–400 cm^−1^ [57]. Both stretching and bending vibration assignments were compared with data from the literature. Figure 5 represents the FTIR spectrum of UD, MC, and MK extracts in the form of KBr pellets and shows the presence of phenols, alcohols, ketones, nitro compounds, esters, carboxylic acids, ethers, aliphatic fluoro, alkenes, and aromatic rings. The broad absorption bands observed at 3358.28, 3327.40, and 3307.88 cm^−1^ were attributed to the stretching of hydroxyl groups and H–bonding in alcohol or phenol groups [58]. The weak absorption peaks in alkanes were detected at 2923.25, 2921.90, and 2974.04 cm^−1^, which correspond to C–H stretching. N–H bends in primary amines were indicated by the high absorption peaks at 1698.70, 1622.77, 1601.79, and 1611.44 cm^−1^. C–C stretching in aromatic groups was assigned to the medium peaks at 1455.85 and 1404.99 cm^−1^. The rocking of the methyl group was assigned to the vibrational absorption bands at 1399.89 and 1396.51 cm^−1^. C-O stretching was represented by distinct bands at 1271.82 and 1275.04 cm^−1^. The C–N stretch in aliphatic amines was assigned to the thin peaks at 1055.84, 1074.42, 1043.61, and 1044.62 cm^−1^. The aromatic H out-of-plane bending had bands at 877.47, 720.06, 659.68, and 620.82 cm^−1^ [59].

The HPLC technique is repeatable, sensitive, and reliable. The existence of 24 chemicals in the ethanolic fractions of UD, MC, and MK was identified using HPLC. In the UD ethanolic extract, three classes of phenols were characterized: anthocyanin compounds (rosinidin 3-O-rutinoside; peonidin 3-O-rutinoside; and peonidin 3-O-6′-O-coumaroyl glucoside), hydroxycinnamic acid derivatives (p-coumaric acid; chlorogenic acid; caffeoylquinic acid; and 2-O-caffeoylmalic acid), and flavonoids (rutin; isorhamnetin 3-O-rutinoside; quercetin; p-coumaroyl glucoside; kaempferol 3-O-rutinoside; and quercetin 3-O-glucoside), as described earlier [38]. The polyphenolic compounds found in the ethanolic fractions of MC flowers were identified by comparing with a previous study [60]. Our findings showed the existence of important constituents, such as quercetin (quercetin-7-O-β-glucoside; quercetin-3-O-β-rutinoside; and quercetin-3-O-β-galactoside), apigenin (apigenin-7-O-7-glucoside; apigenin-7-O-apiosyl-glucoside; and apigenin-7-O-glucosyl-6′-acetate), luteolin (luteolin-7-O-β-glucoside; luteolin-4′-O-7-β-glucoside; and luteolin-7-O-β-rutinoside), isorhamnetin (isorhamnetin-7-O-β-glucoside), patuletin (patuletin-7-O-β-glucoside), eupatoletin, astragalin, and spinacetin [61]. The MK ethanolic fraction was examined by HPLC-DAD, which permitted the identification of important components such as chlorogenic acid, quercitrin, citric acid, piperine, 7 p-coumaric acid, hesperidin, rutin, gallic acid, β-terpineol, ferulic acid, catechin, naringenin, D-α-pinene, di-α-phellandrene, dipentene, D-sabinene, caryophyllene, nicotinic acid, koenigine-quinone A, and koenigine-quinone B [62]. The various phytochemicals in the UD, MC, and MK extracts that are responsible for their antioxidant and protective potential have been well identified. UD has been extensively studied and has shown prominent results in the treatment of prostate enlargement [13], preventing colon carcinogenesis in rats [14], and providing a protective effect against hyperglycemia [15], hypertension [16], and hypercholesterolemia [17]. Numerous studies have shown that MC counteracts diabetes by controlling GLP-1, which is essential for promoting insulin gene transcription [18]. Chamomile oil has also been shown to significantly reduce osteoarthritis [19], and it may be useful in the treatment of lung cancer [20]. Mondal et al., 2022 found that MK modulates various cellular programs and signaling cascades to intervene as an antioxidant in normal cells, as a pro-oxidant in lung carcinoma cells [21], and protects against liver damage caused by TPA [22]. Additionally, earlier research on the MK leaf fraction reported its efficacy in the management of hyperglycemia [23]. Although UD, MC, and MK have been studied in some in vivo and in vitro experiments, there is little evidence for the effect of these three herbs on cellular and neurological complications.

Considering the general protective, organismal, or cognitive effects of UD, MC, and MK that have been reported in the literature, the present study treated third instar larvae and freshly eclosed flies with 500 µM ROT alone or in combination with UD, MC, and MK for 24 to 120 h. Following exposure, cytotoxicity assays (dye exclusion test), biochemical assays (protein estimation and acetylcholinesterase inhibition assays), and behavioral assays (climbing and jumping assays) were performed. ROT is a well-known generator of reactive oxygen species (ROS), which cause cellular damage and eventually lead to necrosis or programmed cell death. Lacking an effective antioxidant system, cells are unable to prevent the harm caused by ROS. *_L_*-DOPA appears to simply act as a dopamine precursor to restore endogenous dopamine deficits, as previous studies have shown that feeding the drug to flies did not reduce cell loss [63].

The cytotoxicity of rotenone and amelioration of cytotoxicity through the use of bioactive compounds (UD, MC, and MK) were determined through a dye exclusion test (trypan blue) in whole larvae and tissues of ROT-exposed organisms. Of the larvae exposed to ROT, 95% showed blue staining in the whole larvae and their tissues (brain ganglia, salivary gland, midgut, and gastric caeca). ROT combined with MK exhibited significantly less blue staining than the ROT + UD and ROT + MC groups in the whole larvae and the abovementioned tissues, respectively. This observation is supported by a previous study on ROT, which found that nutraceuticals significantly improved cell viability [64]. This protective property of UD, MC, and MK may be attributed to the presence of bioactive components responsible for quenching free radicals or due to the upregulation of antioxidative defense mechanisms.

To comprehend the unfavorable effects of ROT that increase cellular oxidant levels and cause proteins to undergo oxidative post-translational modifications, biochemical studies were carried out after giving treatment for 24 and 48 h. Third instar larvae of *D. melanogaster* exposed to 500 µM ROT exhibited a statistically significant decrease (*p* < 0.001) in the total protein content of their tissues. After 24 h, the protein content in the larvae was reduced in the ROT treated group compared with the control and DMSO control group. ROT coexposed with MK showed improved protein concentrations, followed by ROT + UD and ROT + MC. After 48 h, the trend remained the same in comparison with the control group, and the ROT treatment decreased the protein content of the larvae. Increased protein was observed in ROT coexposed with MK, followed by ROT + UD, and lowest in ROT + MC. This finding is consistent with previous research that found various pesticides led to reduced protein content in organisms [65].

Acetylcholinesterase (AChE) is a vital enzyme of the cholinergic system that modulates physiological processes, including memory and locomotor activities. It hydrolyzes acetylcholine to choline and acetate, thereby terminating cholinergic neurotransmission between synapses. In this study, it was found that when the larvae were exposed to ROT for 24 h, they exhibited a statistically significant (*p* < 0.001) inhibition of AChE activity compared with the control or DMSO and had ~60% reduced AChE levels. When ROT was coexposed with MK, AChE levels were improved, and only 9.7% inhibition was evident. AChE levels were improved to a lesser extent in the ROT + UD and ROT + MC groups compared with the control. These elevated levels of AChE were significant when compared with the ROT-treated groups. The maximum inhibition of AChE levels was present in ROT-exposed organisms after 48 h compared with control larvae, and the highest rescue from ROT-induced toxicity was observed in the ROT + MK group, followed by the ROT + UD and ROT + MC groups. We observed that inhibition of AChE in ROT-exposed organisms and nutraceuticals helps to rescue AChE levels, which is supported by previous observations [66,67].

An organism’s behavior reflects its typical physiological activity. Climbing and jumping activities in this context reflect the physiological condition of the organism. Therefore, a high rate of locomotor deficits as evaluated by the climbing assay may indicate rotenone-induced neurotoxicity. Due to their propensity to remain at the base of the plastic tube, flies with locomotor deficits do not appear to have normal leg coordination. This phenotypic expression has previously been attributed to the high energy needs of the muscles used for walking and flying, which are packed with mitochondria. Although speculative, uncoupled mitochondrial machinery may likely be to blame for the same underlying conditions of severe complex I inhibition. Surprisingly, MK > UD > MC were able to significantly rescue flies from worsening locomotor dysfunctions, showing that they may be able to protect by restoring the dopamine pool at the mitochondrial level. This finding supports previous research that showed a strong link between dopamine deficiency and locomotor dysfunction [68]. The adverse effect of the pesticide on the organism was shown by the significant decrease in jumping behavior in exposed organisms, which was followed by an inhibition of AChE activity. Inhibition of AChE activity has previously been described as a sign of poor locomotor activity [69]. All nutraceuticals improved the jumping ability of flies. ROT + MK exhibited the highest rescue, followed by ROT + UD, and ROT + MC. In this context, the isolation of naturally occurring antioxidants have raised interest in plant biomass, which has proven to be rich in compounds. Plants possess the ability to biosynthesize a variety of non-enzymatic antioxidants that can reduce ROS-induced oxidative damage [70,71,72,73].

Taken together, the current study suggests that UV-Vis, FTIR, and HPLC analysis differentiates the extracts of UD, MC, and MK. The comparative account of these medicinal herbs revealed significant variation, which can be used to identify plants that have the most phytoconstituents to be used as phyto remedies for a variety of diseases. Based on our biochemical evidence, we conclude that short-term dietary feeding of UD, MC, and MK to *Drosophila melanogaster* has the propensity to attenuate ROT-induced oxidative stress because of its antioxidative properties and capacity to regulate antioxidant defenses. Additionally, their neuroprotective properties were demonstrated by their potential to significantly alleviate rotenone-induced oxidative stress, enhance locomotion, and restore AChE levels. Moreover, these findings reveal that these plants are medicinally important and should be studied further to locate bioactive compounds and determine their significance in pharmaceutical industries. In the future, we will work with advanced spectroscopic and nanotechnology-based investigations for the identification and structural elucidation of compounds present in UD, MC, and MK against ROT-induced toxicities.

## 5. Conclusions

Current research provides evidence that the antioxidant and antiradical activities of three ethnomedicinal plants collected from different geographical origins and varieties have statistically significant (*p* < 0.001) varied complex chemical mixtures. This investigation has provided preliminary information to determine the chemical composition of *U. dioica, M. chamomilla,* and *M. koenigii* using UV-Vis, FT-IR, and HPLC techniques. From the above investigations, it can be concluded that MK has higher anti-radical activity in aqueous as well as ethanolic extracts in DPPH, ABTS, TPC, and TFC assays among the screened plants. The presence of the O-H, C-H, N-H, C-O, C=O, C-C, C-N, N=C, S=O, and C=N groups were predicted by UV-Vis, FT-IR, and HPLC. Additionally, we conclude that short-term nutritional feeding of UD, MC, and MK to *Drosophila* has the potential to reduce ROT-induced oxidative stress due to its antioxidative properties and capacity to regulate antioxidant defense mechanisms. They are promising plants for future research due to their antioxidant capabilities and might help slow or prevent the process of oxidative stress-related diseases. The findings of this study will be useful in the quality control of raw herbaceous material to verify their potential for phytopharmaceutical applications and health-promoting properties that could be used in drug discovery. However, further research is needed for a better understanding of their bioactivity, toxicity profile, and impact on the ecosystem and agricultural commodities.

## Figures and Tables

**Table 1:** The percentage yield of plant extracts using aqueous and ethanolic extraction methods.

S. No.	Plant Species	Code	Plant Part Used	Solvents (%) Yield (*w*/*w*)	
				Aqueous Extracts	Ethanolic Extracts
1.	*U. dioica*	UD	Leaves	14.34	10.45
2.	*M. chamomilla*	MC	Flower	8.56	14.68
3.	*M. koenigii*	MK	Leaves	21.62	15.27

**Table 2:** Estimated EC_50_ (mg/mL) of UD, MC, and MK were obtained by different models using DPPH and ABTS assays.

Assays	Plant Species	Plant Part Used	EC_50_ (mg/mL) of Aqueous Extracts	EC_50_ (mg/mL) of Ethanolic Extracts
DPPH	UD	Leaves	0.42	0.16
	MC	Flowers	1.00	0.12
	MK	Leaves	0.33	0.10
ABTS	UD	Leaves	0.55	0.10
	MC	Flowers	1.04	0.16
	MK	Leaves	0.51	0.07

**Table 3:** TPC and TFC results of UD, MC, and MK are expressed in mean ± SD.

Plant Species	Plant Part Used	TPC mg (GAE)/g (Aqueous)	TPC mg (GAE)/g (Ethanolic)	TFC mg (QE)/g (Aqueous)	TFC mg (QE)/g (Ethanolic)
*U. dioica*	Leaves	26.08 ± 2.02	42.16 ± 2.06	5.458 ± 2.3	12.48 ± 1.04
*M. chamomilla*	Flowers	24.01 ± 1.50	40.5 ± 4.04	5.465 ± 1.06	12.64 ± 2.3
*M. koenigii*	Leaves	35.14 ± 3.0	48.93 ± 2.03	9.641 ± 2.5	22.88 ± 1.05

**Table 4:** Preliminary qualitative screening of secondary metabolites of crude extracts of UD, MC, and MK.

S. No.	Phytoconstituents	Tests	Positive Results	UD	MC	MK
1.	Phenols	Ferric chloride test	Bluish-green	+	+	+
2.	Flavonoids	Alkaline reagent test	Orange-red	+	+	+
3.	Alkaloids	Wagner’s test	Red precipitate	+	+	+
4.	Tannins	FeCl_3_ test	Black blue	+	+	+
5.	Carbohydrates	Molisch’s test	Red or dull violet	+	−	+
6.	Saponins	Foam test	White precipitate	+	+	+
7.	Terpenoids	Salkowski test	Change from pink to violet	+	+	+
8.	Steroids	Liebermann’s test	violet to blue or green color	+	+	+
9.	Glycosides	Keller-Killiani test	Brick red	−	+	+

Note: The presence of phytoconstituents is indicated by a ‘+’ sign, whereas the lack of phytoconstituents is indicated by ‘−’.

**Table 5:** FT-IR frequency range and functional groups are present in the extracts of UD, MC, and MK.

S. No.	Frequency Range (cm^−1^)			Functional Groups	Phytocompounds Identified
	UD	MC	MK		
1.	3358.28	3327.40	3307.88	H-bonded, OH stretching	Hydroxyl compounds
2.	2923.25	2921.90	2974.04	Asymmetric stretching –CH(CH_2_) vibration	Saturated aliphatic Compounds (Lipids)
3.	2853.54	2802.86	2804.74	Symmetric stretching –CH_2_(CH_2_) vibration	Proteins, lipids
4.	1698.70, 1622.77	1601.79	1611.44	C=O stretching vibration	Ketone compound
5.	1455.85	1404.99	-	C=C-C aromatic ring stretching	Aromatic compound
6.	1399.89	-	1396.51	O-H, alcoholic group	Phenol or tertiary alcohol
7.	-	1271.82	1275.04	CN stretching	Aromatic primary amine
8.	1160.88	1174.09	1140.63	Polymeric OH, C-O stretching	Cyclic ether
9.	1055.84	1074.42, 1043.61	1044.62	Phosphate ion	Phosphate compound
10.	-	-	877.47	*p*-O-C stretching	Aromatic phosphate
11.	720.06	620.82	659.68	C-Cl stretching	Aliphatic chloro compound

**Table 6:** Major phytochemical compounds identified in ethanolic extracts *of U. dioica, M. chamomilla*, and *M. koenigii*.

S. No	Retention Time (R_t_ min.)			Compound	Molecular Formulae	Chemical Structure	Molecular Weight (g/mol)	Pharmacological Actions
	UD	MC	MK					
1.	1.931	-	1.645	Fumaric acid	C_4_H_4_O_4_	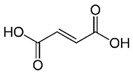	116.07	Reduces gallstone formation, used for the treatment of multiple sclerosis and psoriasis.
2.	4.240	4.235	4.276	Gallic acid	C_7_H_6_O_5_	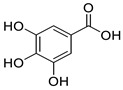	170.12	Expectorant, cytotoxic steroid, memory enhancer, anti-inflammatory, anti-neoplastic, and antioxidant properties.
3.	-	7.523	7.473	Protocatechuic acid	C_7_H_6_O_4_	* 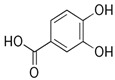 *	154.12	Neuroprotective, antioxidant, anticancer, antibacterial, anti-aging, and anti-asthma properties.
4.	9.211	-	9.865	Catechins	C_15_H_14_O_6_	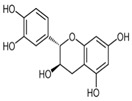	290.27	Used to prevent and treat various diseases, high antioxidant activity, and used in cosmetics.
5.	-	11.387	-	4-O-Caffeoylquinic acid	C_16_H_18_O_9_	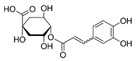	354.31	Cytoprotective, neuroprotective, and hepatoprotective effects.
6.	15.982	14.683	15.554	Caffeic acid derivative	C_9_H_8_O_4_	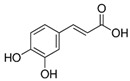	180.16	Prevents DNA damage and oxidative stress induced by free radicals.
7.	16.683	16.299	16.693	Epicatechin	C_15_H_14_O_6_	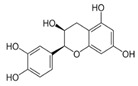	290.27	Reduces blood glucose levels in diabetic patients and stimulates mitochondrial respiration.
8.	17.248	17.558	17.280	Rutin	C_27_H_30_O_16_	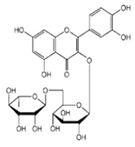	610.5	Hypolipidemic, anti-protozoal, vasoactive, cytoprotective, anti-allergic, anti-platelet, anti-hypertensive, and anti-spasmodic properties.
9.	17.515	17.428	17.625	Syringic acid	C_9_H_10_O_5_	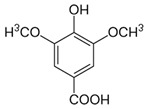	198.17	Used in the prevention of CVDs, cancer, diabetes, and possesses antioxidant activities.
10.	18.094	18.763	18.651	Isorhamnetin-3-O-glucoside	C_22_H_22_O_12_	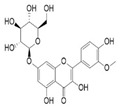	478.4	Anti-viral, antioxidant, anticancer, anti-tumor, anti-inflammatory, and antimicrobial properties.
11.	-	19.243	-	Apigenin-7-O-glucoside	C_21_H_20_O_10_	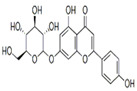	432.4	Prominent chemopreventive, anti-candidal effect, antifungal potential, and strengthens the failing heart.
12.	20.728	20.152	20.835	p-coumaric acid	C_9_H_8_O_3_	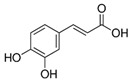	164.16	Anti-inflammatory, antimicrobial, anti-viral, and antibacterial properties.
13.	-	21.739	-	4,5-O-dicaffeoylquinic acid	C_25_H_24_O_12_	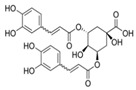	516.4	In melanocytes, significantly reduces tyrosinase activity and melanin synthesis in a dose-dependent manner.
14.	22.564	22.571	22.677	2-O-Caffeoylmalic acid	C_13_H_12_O_8_	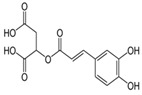	296.230	Prevents ROS production and possesses high antioxidant activity.
15.	23.232	23.924	23.232	Ferulic acid	C_10_H_10_O_4_	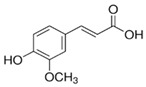	194.18	Wide range of therapeutic uses against various diseases including cancer, arthritis, etc.
16.	-	24.579	-	Naringin	C_27_H_32_O_14_	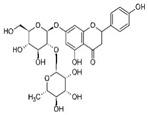	580.5	Anti-carcinogenic and acts as inhibitor of selected cytochrome P_450_ enzymes.
17.	25.247	25.460	25.814	Quercetin (quercetin-3-O-rhamonoside)	C_21_H_20_O_11_	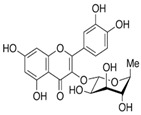	448.4	Used in the treatment of inflammatory, allergic, and metabolic disorders and act as anti-protozoal.
18.	27.723	27.787	27.379	Myricetin	C_15_H_10_O_8_	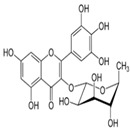	318.23	Acts as an anti-epileptic, anti-amyloidogenic, anti-diabetic, antioxidant, antibacterial, anti-ulcer, antiviral, anticancer, and anti-inflammatory agent.
19.	28.231	28.789	29.031	Quercetin	C_21_H_20_O_11_	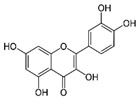	448.4	Decreases tumor necrosis factor (TNF-α) production in macrophages and LPS-driven IL-8 synthesis in lung A549 cells generated by lipopolysaccharide (LPS).
20.	32.156	32.956	31.456	Kaempferol	C_15_H_10_O_6_	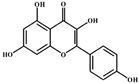	286.24	Anxiolytic, anti-diabetic, anti-estrogenic, anti-osteoporotic, cardioprotective, and neuroprotective properties.
21.	-	34.320	-	Luteolin	C_15_H_10_O_6_	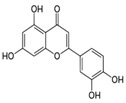	286.24	Exhibits anti-inflammatory properties due to ability to regulate transcription factors like NF-B, AP-1, and STAT3.
22.	-	34.745	-	Cirsiliol	C_17_H_14_O_7_	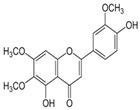	330.29	Act as inhibitor of arachidonate 5-lipoxygenase and has anticancer, hypnotic, sedative, and anti-inflammatory properties.
23.	35.339	35.445	36.021	Isorhamnetin	C_16_H_12_O_7_	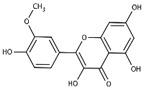	316.26	Cerebrovascular and cardiovascular protective properties; in addition, has antioxidant, anti-tumor, anti-inflammatory, organ protection, and obesity prevention properties.
24.	37.277	-	37.552	Kaempferol 3-O-glucoside	C_21_H_14_O_11_	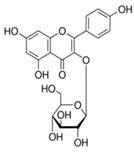	448.38	Lowers the risk of chronic diseases, particularly cancer, and boosts the body’s antioxidant defenses against free radicals.
25.	39.901	41.026	40.516	Isorhamnetin 3-O-rutinoside	C_28_H_32_O_16_	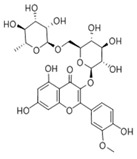	624.5	Inhibits membrane proteins and has anti-apoptosis, antioxidation, anti-tumor, anti-inflammation, antiviral, antibacterial, anti-amyloidogenic, and anti-diabetic properties.

## Data Availability

Data are contained within the article.
